# New records of the genus *Aleiodes* (Hymenoptera, Rogadinae) from South Korea

**DOI:** 10.3897/BDJ.13.e170343

**Published:** 2025-12-01

**Authors:** Moonhwan Yu, Sangjin Kim, Juhyeong Sohn, Hyojoong Kim

**Affiliations:** 1 Kunsan National University, Gunsan-si, Republic of Korea Kunsan National University Gunsan-si Republic of Korea

**Keywords:** DNA barcoding, parasitoid wasps, taxonomy, unrecorded species

## Abstract

**Background:**

The subfamily Rogadinae comprises several genera, amongst which *Aleiodes* Wesmael, 1838 is the most species-rich, with more than 632 species described worldwide and many others likely remaining undiscovered.

**New information:**

In this study, we report three species of *Aleiodes* newly recorded from South Korea: *Aleiodes angustipterus* van Achterberg & Shaw, 2016, *Aleiodes euproctis* He & Chen, 1990 and *Aleiodes malichi* Butcher et al., 2012.

## Introduction

The Rogadinae (Hymenoptera: Braconidae) comprises a distinctive group of parasitoid wasps known for their specialized role as endoparasitoids of Lepidopteran larvae. Most members of Rogadinae are koinobionts, allowing their hosts to continue developing for a period after parasitism before being ultimately killed. A characteristic trait of many Rogadinae species is their ability to induce host mummification, in which the host’s integument hardens and forms a protective casing for the developing wasp pupa (Van Achterberg 1991). This mummification behaviour is a key diagnostic feature of the group and is considered an adaptive trait enhancing offspring survival.

The subfamily currently includes 63 genera (Shimbori et al. 2024), but *Aleiodes* Wesmael, 1838 (Wesmael 1838) is by far the most species-rich and widely distributed. Within Rogadinae, *Aleiodes* represents a particularly successful radiation, with over 632 described species and numerous others likely remaining undiscovered (Yu et al. 2016). The genus occurs across all biogeographical regions and shows especially high species richness in tropical and subtropical forests (Townsend and Shaw 2009). *Aleiodes* species are ecologically significant as parasitoids of Lepidopteran larvae and some have been investigated for their potential as biological control agents, although such applications remain limited.

Taxonomically, *Aleiodes* is one of the most morphologically diverse and complex genera in the family, characterised by a combination of diagnostic features, such as host mummification, the absence of a prepectal carina, a relatively short ovipositor and distinctive wing venation (Van Achterberg 1991). Despite considerable taxonomic work on the genus, many undescribed or cryptic species continue to be discovered worldwide and the relationships amongst species remain insufficiently resolved. Integrative approaches combining morphological and molecular data are, therefore, essential to clarify phylogenetic relationships and species limits within the genus.

In South Korea, the fauna of *Aleiodes* has received comparatively little attention and available records indicate that the distributional and taxonomic information on this genus remain insufficient. Currently, 36 species of *Aleiodes* have been recorded from South Korea (He 1997, Belokobylskij 2000, Van Achterberg and Shaw 2016, Yu et al. 2016, National Institute of Biological Resources 2024). Previous studies on Korean braconids have primarily focused on other subfamilies, leaving the diversity of *Aleiodes* still incompletely documented.

In this study, we report three *Aleiodes* species newly recorded from South Korea and provide their descriptions, diagnoses, distribution data, illustrations and DNA barcode sequences. These data contribute to the growing knowledge of *Aleiodes* diversity in East Asia and highlight the need for continued faunistic and integrative taxonomic studies on the Korean Peninsula.

## Materials and methods

### Field and Taxonomic works

All specimens of the studied species were collected using Malaise traps. The collected samples were preserved in 80% ethyl alcohol. All specimens are deposited in the insect collection of Kunsan National University (KSNU), South Korea. Morphological terminology follows van Achterberg (Van Achterberg 1988, Van Achterberg and Shaw 2016).

Observations and photographs were made using a LEICA M205 C stereomicroscope, equipped with a LEICA DMC2900 digital camera (Leica Geosystems AG). Image stacks were processed with Helicon Focus software to obtain extended depth of field.

### Molecular works

DNA was extracted using the LaboPass Tissue Mini DNA Isolation Kit (COSMO Genetech, Daejeon, Korea) following the manufacturer’s protocol with slight modifications. Each specimen was incubated in 200 μl of lysis buffer and 20 μl of proteinase K solution at 55°C for 3 hours and then stored at –22°C for 12–24 hours.

The mitochondrial *COI* gene was amplified using primers LCO-1490 (5’-GGTCAACAAATCATAAGATATTGG-3’) and HCO-2198 (5’-TAAACTTCAGGTGACCAAAAAATCA-3’) (Vrijenhoek 1994). PCR was performed in 20 μl reaction mixtures containing 0.4 μM of each primer, 200 μM of each dNTP, 2.0 mM MgCl₂ and approximately 50 ng of genomic DNA, using AccuPower PCR PreMix (BIONEER, Daejeon, Korea) on a GS1 thermal cycler (Gene Technologies Ltd., Essex, UK).

Polymerase chain reaction (PCR) was performed with an initial denaturation at 95°C for 5 minutes, followed by 34 cycles of denaturation at 94°C for 35 seconds, annealing at 48°C for 25 seconds and extension at 72°C for 45 seconds. A final extension was conducted at 72°C for 5 minutes. PCR products were visualised as a single band by electrophoresis on a 1.5% agarose gel. Amplified products were purified using the QIAquick PCR Purification Kit (QIAGEN, Inc., Milan, Italy) and subsequently sequenced using an automated sequencer at Macrogen Inc (Seoul, South Korea).

## Taxon treatments

### Aleiodes angustipterus

van Achterberg & Shaw, 2016

DA313661-2A5C-5939-B53E-FEF92BBFA2D3

PV988353

#### Materials

- **Type status:** Other material. **Occurrence:** sex: female; lifeStage: adult; occurrenceID: 0217A57F-FF1D-5706-ABAA-66ED481679C1; **Location:** country: South Korea; countryCode: KR; stateProvince: Incheon; municipality: Ganghwa-gun; locality: Naega-myeon; **Event:** verbatimEventDate: 24 Sep 2020; **Record Level:** institutionCode: NIBR, NATIONAL INSTITUTE OF BIOLOGICAL RESOURCES; **Material Entity:** associatedSequences: https://www.ncbi.nlm.nih.gov/nuccore/PV988353.1/

#### Description

**Figure 1.**
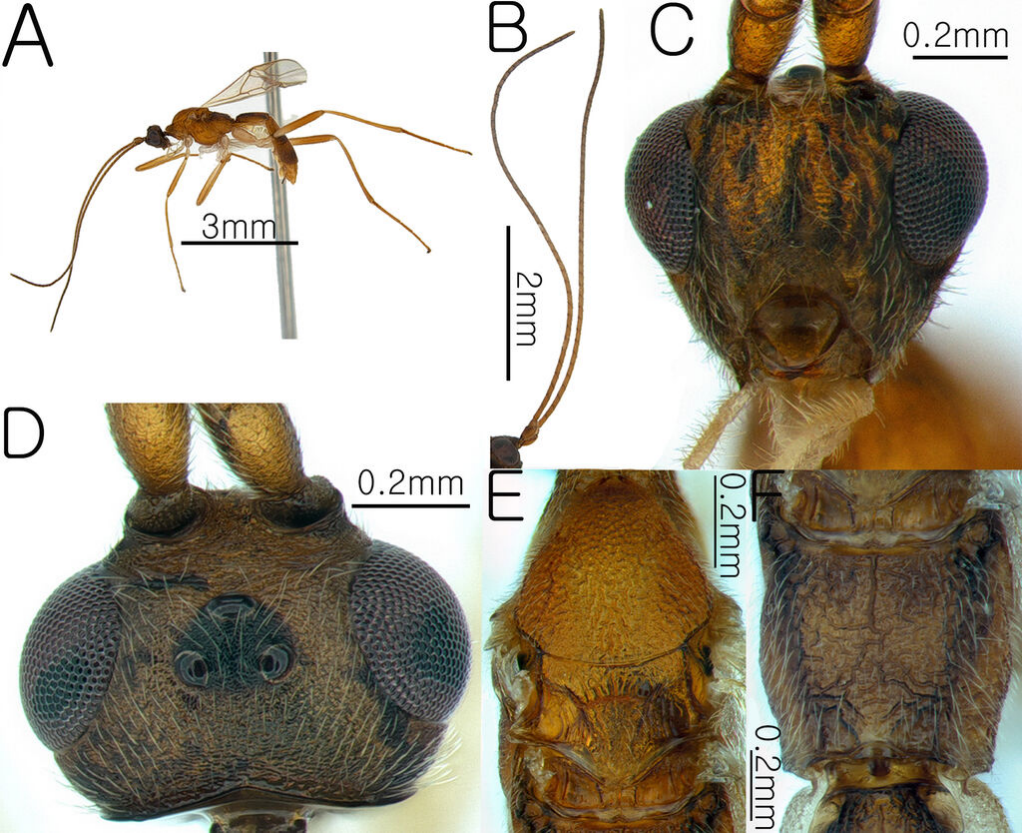
Habitus of *Aleiodes angustipterus* van Achterberg & Shaw, 2016. **A** whole body in lateral view; **B** antenna; **C** head in anterior view; **D** head in dorsal view; **E** mesosoma in dorsal view; **F** propodeum in dorsal view.

**Figure 2.**
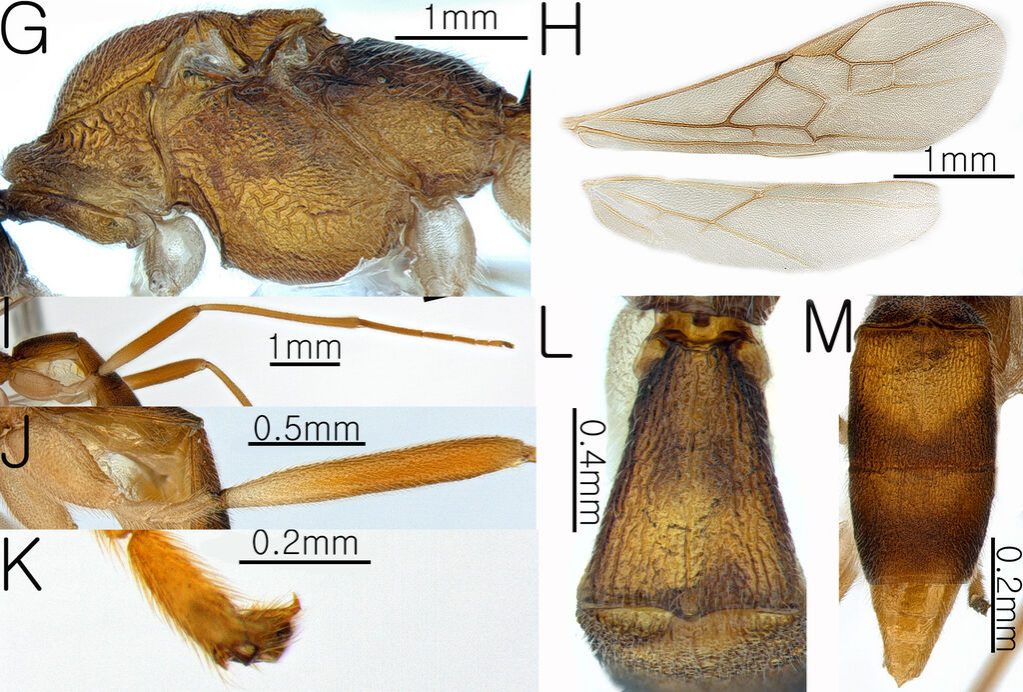
Habitus of *Aleiodes angustipterus* van Achterberg & Shaw, 2016. **G** mesosoma in lateral view; **H** wings; **I** hind leg in lateral view; **J** hind coxa and femur in lateral view; **K** hind leg tarsal claw; **L** first metasomal tergite in dorsal view; **M** second and third metasomal tergite in dorsal view.

**Female. Body**. Length of body 4.8 mm, length of fore-wing 3.7 mm (Fig. 1A and Fig. 2H).

-**Head**. Antennae dark brown; length of antennae 5.8 mm, with 46 flagellomeres; apical segments pointed (Fig. 1B), frons granulate and slightly shiny; OOL and POL 1.48× and 1.4× the width of the posterior ocellus; vertex granulate-coriaceous; length of eye 2.35× length of temple; occipital carina complete and arched (Fig. 1D); width of hypoclypeal depression 0.4× width of the face (Fig. 1C).

**Mesosoma.** Mesoscutal lobes coriaceous and matt; notauli narrow and weakly present; scutellum rugose and yellowishbrown (Fig. 1E); propodeum rugose, with the median carina present only in the anterior half (Fig. 1F); prepectal carina complete, reaching the anterior margin; area below the precoxal sulcus of the mesopleuron reticulate-rugose (Fig. 2G).

**Wings.** Fore-wing: pterostigma yellowish-brown; vein r 0.4 × 3-SR; 1-CU1 0.43 × 2-CU1; vein r-m 0.72 × 2-SR and 0.43 3-SR; vein cu-a straight and not parallel to vein CU1b; vein 1-M straight, 1-SR forms an angle with it; Hind-wing: apical half of marginal cell gradually widened posteriorly; vein m-cu present and short (Fig. 2H).

**Leg.** Hind leg tarsal claws yellow bristles and pecten absent (Fig. 2K); hind coxa 0.5× length of first metasomal tergite; length of fore and hind femur 9.0× and 8.6× their respective widths (Fig. 2I and Fig. 2J); comb on inner apex of hind tibia absent.

**Metasoma.** Length of first metasomal tergite 1.05× apical width of first metasomal tergite (Fig. 2L); first and second metasomal tergite longitudinally rugose and median carina present (Fig. 2L and Fig. 2M); third metasomal tergite longitudinally rugose and median carina present only in the basal half; second and third metasomal tergites with a yellowish-brown pentagonal mark (Fig. 2M); ovipositor sheath densely setose.

#### Diagnosis

Eyes small, with the length of the eye 2.35× the length of the temple; occipital carina complete and distinctly arched; notauli weak and narrow; propodeum rugose, with median carina present; fore-wing vein 1-M straight; hind coxa 0.5x shorter than first metasomal tergite; first metasomal tergite 1.05× as long as its apical width; and second metasomal tergite with a distinct yellowish-brown pentagonal mark. Morphologically similar to *Aleiodes jakowlewi*, but in *A. jakowlewi*, the hind coxa is nearly as long as the first metasomal tergite and the second metasomal tergite is comparatively long. In contrast, *A. angustipterus* has the hind coxa 0.5× shorter than the first metasomal tergite and the second metasomael tergite is comparatively short. The pterostigma of *A. jakowlewi* is dark brown with the basal third paler, whereas, in *A. angustipterus*, it is entirely dark brown. The eye is relatively large in *A. jakowlewi*, but small in the present species. The female antenna of *A. jakowlewi* occasionally bears a narrow pale whitish or yellowish submedial band, which is absent in *A. angustipterus* (Van Achterberg and Shaw 2016).

#### Distribution

South Korea (new record), Europe, China, Japan.

#### Notes

The Korean specimens closely correspond to the original description of *Aleiodes angustipterus* (Van Achterberg and Shaw 2016), but exhibit several minor morphological differences. The antennae of the Korean specimens comprise 46 flagellomeres, whereas the type material was described with 36–40. The OOL is 1.48× the width of the posterior ocellus, compared with 1.2× in the original description. The pterostigma is yellowish-brown rather than dark brown as originally noted and the mesosoma and metasomal tergites are generally lighter brown than in the uniformly dark brown type specimens.

#### Parasite of

*Hypenodes humidalis* (Van Achterberg and Shaw 2016).

### Aleiodes euproctis

He & Chen, 1990

748338B8-E677-5311-A36C-F862E4C81437

PV988353

#### Materials

- **Type status:** Other material. **Occurrence:** sex: 1 female; lifeStage: adult; occurrenceID: D622AB32-59D9-5765-A7E9-756F6CEC6B86; **Location:** country: South Korea; countryCode: KR; stateProvince: Chungcheongbuk-do; municipality: Boeun-gun; locality: (Minpan-dong) Songnisan; **Event:** verbatimEventDate: 15 Oct 2022; **Record Level:** institutionCode: NIBR, NATIONAL INSTITUTE OF BIOLOGICAL RESOURCES; **Material Entity:** associatedSequences: https://www.ncbi.nlm.nih.gov/nuccore/PV988354

#### Description

**Figure 3.**
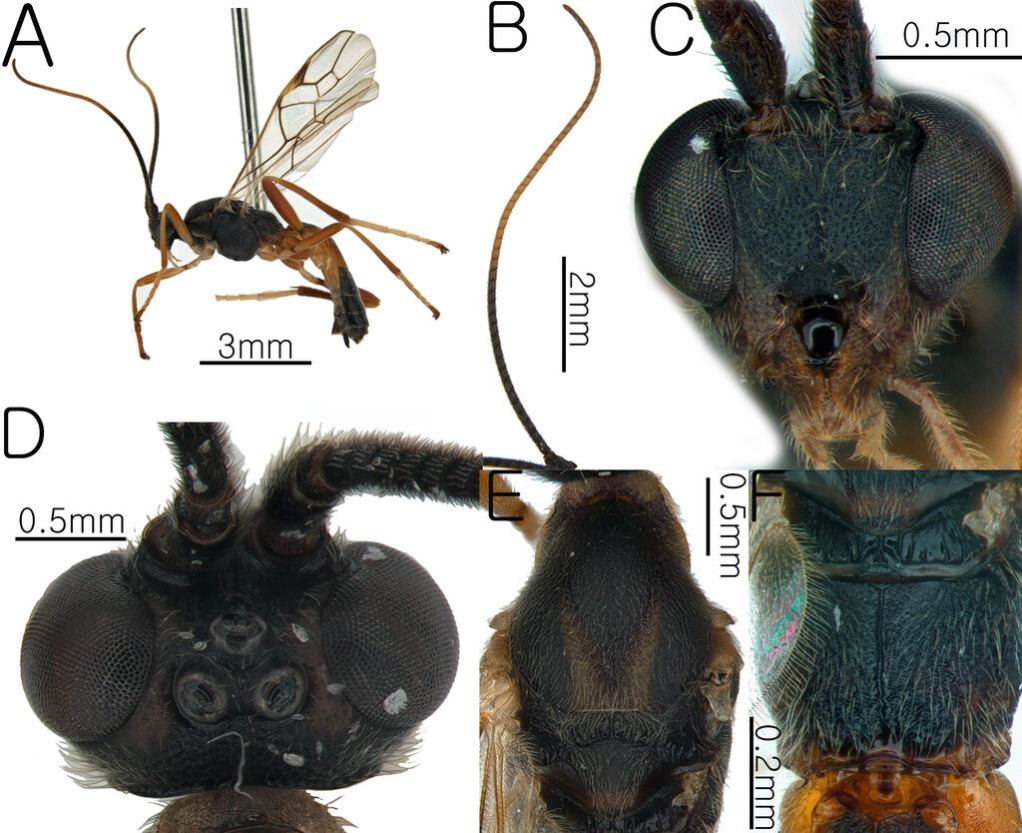
Habitus of *Aleiodes euproctis* Chen & He, 1990. **A** whole body in lateral view; **B** antenna; **C** head in anterior view; **D** head in dorsal view; **E** mesosoma in dorsal view; **F** propodeum in dorsal view.

**Figure 4.**
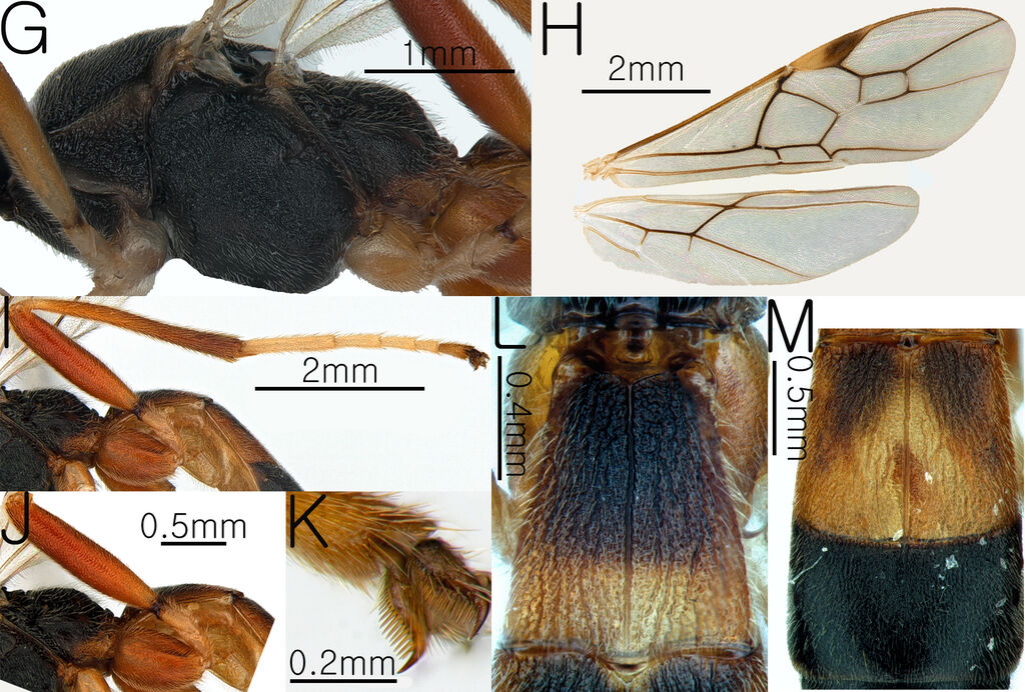
Habitus of *Aleiodes euproctis* Chen & He, 1990. **G** mesosoma in lateral view; **H** wings; **I** hind leg in lateral view; **J** hind coxa and femur in lateral view; **K** hind leg tarsal claw; **L** first metasomal tergite in dorsal view; **M** second and third metasomal tergite.

**Female Body**. Length of body 7.1 mm, length of fore-wing 6.27 mm (Fig. 3A and Fig. 4H).

**Head**. Basal half of the antennae blackish, apical half yellowish-brown; length of antennae 7.9 mm, with 50 flagellomeres; apical segments pointed (Fig. 3B); frons granulate and flat (Fig. 3C); OOL and POL 0.45× and 0.47× the width of the posterior ocellus; vertex coriaceous; length of eye 2.62 × length of temple; occipital carina complete and weakly arched (Fig. 3D); width of hypoclypeal depression 0.3× width of the face (Fig. 3C).

**Mesosoma.** Mesoscutal lobes coriaceous and matt; notauli yellowish-brown, broad and distinctly present; scutellum coriaceous-rugulose and posteriorly yellowish-brown (Fig. 3E); propodeum rugose, with the median carina present in the posterior margin (Fig. 3F); prepectal carina complete, reaching the anterior margin; area below the precoxal sulcus of mesopleuron coriaceous-rugulose (Fig. 4G).

**Wings.** Fore-wing: pterostigma dark brown, with apex pale yellow; vein r 0.49 × 3-SR; 1-CU1 0.46 × 2-CU1; vein r-m 0.78 × 2-SR and 0.36 3-SR; vein cu-a straight and parallel to vein CU1b; vein 1-M weakly curved; vein 1-SR dark brown, forming an angle with 1-M; Hind-wing: apical half of marginal cell slightly widened posteriorly; vein m-cu present (Fig. 4H).

**Leg.** Hind leg tarsal claws brown and yellowish pecten present (Fig. 4K); hind coxa 0.55× length of first metasomal tergite; length of fore and hind femur 5.62× and 6.26× their respective widths (Fig. 4I and Fig. 4J); comb on inner apex of hind tibia absent.

**Metasoma.** Length of first metasomal tergite 1.02× apical width of first metasomal tergite (Fig. 4L); first and second metasomal tergite longitudinally rugose and median carina present (Fig. 4L and Fig. 4M); third metasomal tergite rugulose and median carina short present; basal half of the first metasomal tergite black; second metasomal tergite with black triangular mark on the dorsolateral surface (Fig. 4M); ovipositor sheath moderately setose.

#### Diagnosis

Basal half of the antenna blackish and apical half yellowish-brown, ocellus large; precoxal sulcus distinct; body predominantly blackish-brown with yellowish spots on the head and mesosoma; pterostigma dark brown, with apex pale yellow; hind tarsal claws yellowish, with a distinct pecten; basal half of the first metasomal tergite black; and second metasomal tergite bearing a distinct black triangular mark. *Aleiodes euproctis* is similar to *Aleiodes apiculatus* in general body colouration and wing venation, but can be distinguished by the following combination of characters. The antenna of *A. euproctis* consists of 50 flagellomeres, whereas *A. apiculatus* has 46-49 segments. In *A. euproctis*, the basal half of the antenna is blackish and the apical half is yellowish, while in *A. apiculatus*, the antenna is mostly yellowish medially with dark brown colouration at the base and apex. The notauli in *A. euproctis* are yellowish-brown, broad and distinctly developed, whereas those in *A. apiculatus* are narrow and weakly defined. The hind femur of *A. euproctis* is longer and more slender, measuring 6.26× its maximum width, compared to approximately 4.5× in *A. apiculatus*. The hind coxa of *A. euproctis* is shorter than the first metasomal tergite, measuring approximately 0.55× its length, while in *A. apiculatus*, the hind coxa is nearly equal in length to the first tergite. A distinct black triangular mark is present on the dorsolateral surface of the second metasomal tergite in *A. euproctis*, which is absent in *A. apiculatus*. Furthermore, the ovipositor sheath is moderately setose in *A. euproctis*, whereas it is densely setose *in A. apiculatus* (He 1997, Van Achterberg and Shaw 2016).

#### Distribution

South Korea (new record), China.

#### Notes

According to He 1997, the body length of *Aleiodes euproctis* is 5–6 mm, whereas the Korean specimens measure 7.9 mm. The length of the eye is 2.62× the length of the temple in the Korean specimens, compared with 3.5–6.2× in the original description. This species had not been previously registered in NCBI and the present study provides the first *COI* sequence for *A. euproctis*.

#### Parasite of

*Euproctis bipunctapex* (He and Chen 1990 , He 1997).

### Aleiodes malichi

Quicke & Butcher, 2012

FA5D8A70-2E56-5F12-96EF-20FB4EEDC1B2

PV988355

#### Materials

- **Type status:** Other material. **Occurrence:** sex: 1 female; lifeStage: adult; occurrenceID: 9D8FFF60-1385-510B-9591-1B268C8F643E; **Location:** country: South Korea; countryCode: KR; stateProvince: Gangwon-do; county: Wonju-si; municipality: Socho-myeon; locality: 55, Guryongmaeul-gil; **Event:** endDayOfYear: 24 Jun 24; **Record Level:** institutionCode: NIBR, NATIONAL INSTITUTE OF BIOLOGICAL RESOURCES; **Material Entity:** associatedSequences: https://www.ncbi.nlm.nih.gov/nuccore/PV988355

#### Description

**Figure 5.**
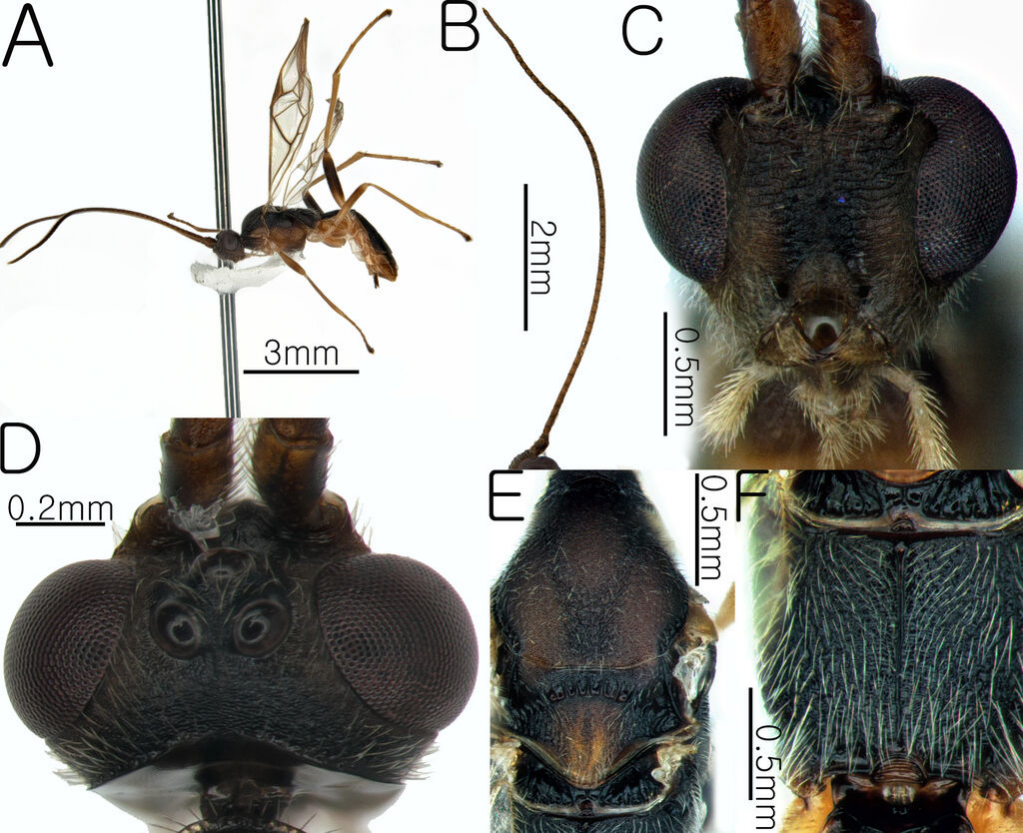
Habitus of *Aleiodes malichi* Quicke & Butcher, 2012. **A** whole body in lateral view; **B** antenna; **C** head in anterior view; **D** head in dorsal view; **E** mesosoma in dorsal view; **F** propodeum in dorsal view.

**Figure 6.**
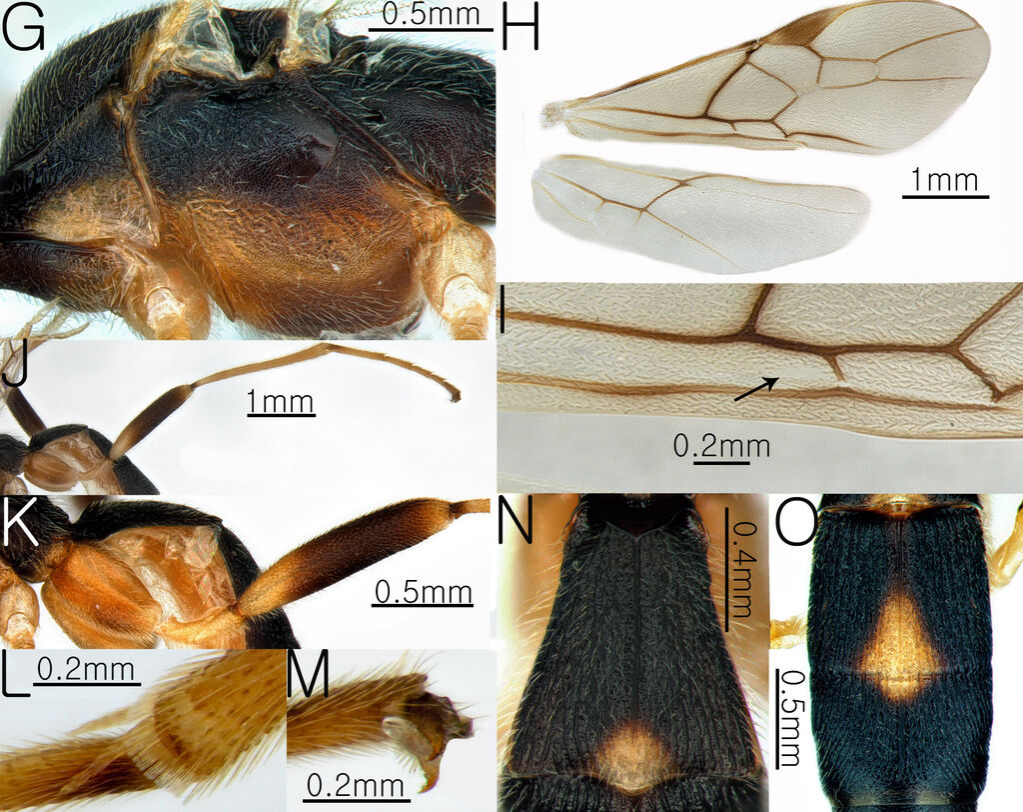
Habitus of *Aleiodes malichi* Quicke & Butcher, 2012. **G** mesosoma in lateral view; **H** wings; **I** sub-basal cell of fore-wing; **J** hind leg in lateral view; **K** hind coxa and femur leteral view; **L** inner apex of hind tibia; **M** hind tarsal claw; **N** first metasomal tergite in dorsal view; **O** second and third metasomal tergite in dorsal view.

**Female Body.** Length of body 5.8 mm, length of fore-wing 6.9 mm (Fig. 5A and Fig. 6H).

**Head.** Antennae dark brown; length of antennae 6.9 mm, with 47 flagellomeres; apical segments pointed (Fig. 5B); frons rugose and flat (Fig. 5C); OOL and POL 0.39× and 0.45× the width of the posterior ocellus; vertex coriaceous; length of eye 3.48× length of temple; occipital carina slightly interrupted and arched (Fig. 5D); width of hypoclypeal depression 0.3× width of the face (Fig. 5C).

**Mesosoma.** Mesoscutal lobes coriaceous and matt; notauli yellowish dark, broad and distinctly present; scutellum coriaceous and entirely yellowish-brown (Fig. 5E); propodeum rugulose, with the median carina present in the posterior margin (Fig. 5F); prepectal carina complete, reaching the anterior margin; area below the precoxal sulcus of mesopleuron coriaceous-rugose (Fig. 6G).

**Wings.** Fore-wing: pterostigma dark brown, with apex pale yellow; vein r 0.36 × 3-SR; 1-CU1 0.64 × 2-CU1; vein r-m 0.62 × 2-SR and 0.28 3-SR; vein cu-a straight and not parallel to vein CU1b; vein 1-M weakly curved; vein 1-SR dark brown, forming an angle with 1-M; Sub-basal cell apically with a glabrous area (Fig. 6I); Hind-wing: apical half of marginal cell gradually widened posteriorly; vein m-cu present (Fig. 6H).

**Leg.** Tarsal claws brown and yellowish small pecten present (Fig. 6M); hind coxa 0.81× length of first metasomal tergite; length of fore and hind femur 7.26× and 5.38× their respective widths (Fig. 6J and Fig. 6K); comb on inner apex of hind tibia present (Fig. 6L).

**Metasoma**. Length of first metasomal tergite 1.07× apical width of first metasomal tergite (Fig. 6N); first and second metasomal tergite longitudinally rugose and median carina present (Fig. 6N and Fig. 6O); third metasomal tergite rugose and median carina present; first and second metasomal tergites posteriorly with yellowish-brown triangular markings (Fig. 6O); ovipositor sheath moderately setose.

#### Diagnosis

Antennae with 47 flagellomeres; occipital carina slightly interrupted and arched; precoxal sulcus distinct; propodeum with the median carina present; fore-wing sub basal cell apically with a glabrous area; hind-wing vein m-cu distinct; inner apex of hind tibia with a comb of adpressed setae; tarsal claws yellowish, with a small pecten; and third metasomal tergite with a distinct median carina. The mesopleuron of *Aleiodes seriatus* is punctulate; in *Aleiodes malichi*, it is coriaceous-rugose. In the fore-wing, *A. seriatus* has vein r 0.4× the length of 3-SR and vein 2-CU1 1.3× the length of 1-CU1; in *A. malichi*, vein r is 0.36× 3-SR and vein 2-CU1 is 1.56× 1-CU1. The marginal cell of the hind-wing is apically parallel-sided in *A. seriatus*, but gradually widened posteriorly in *A. malichi*. The claws of *A. seriatus* are simple (Farahani et al. 2015), in contrast to *A. malichi*, which has a small yellowish pecten (Butcher et al. 2012).

#### Distribution

South Korea (new record), Thailand.

#### Notes

The antennae are slightly longer (6.9 mm vs. 5.7 mm in the original description), although both possess 47 flagellomeres. The fore-wing vein ratios differ slightly (2-CU1 : 1-CU1 = 1.56× in the Korean specimens vs. 1.05× in the original description). The mesosoma and metasoma are also somewhat lighter in colour, with yellowish-brown markings on the scutellum and second tergite, compared with the uniformly dark brown colouration of the type material.

#### Parasite of

Unknown.

## Identification Keys

### Key to species of *Aleiodes* Wesmael recorded from Korea

**Table:** 

1	Mesopleuron distinctly coriaceous-rugose, strongly longitudinally striate dorsally; tarsal claws yellowish with a small pecten; hind tibia with comb of adpressed setae	***Aleiodes malichi* Quicke & Butcher, 2012**
–	Mesopleuron punctulate or finely rugulose, not longitudinally striate; tarsal claws simple, without pecten; hind tibia with comb of adpressed setae	***Aleiodes seriatus* Herrich-Schäffer, 1838**
2	Antenna bicoloured, basal half blackish and apical half yellowish-brown; notauli broad and distinct; body blackish-brown with yellowish spots; second metasomal tergite bearing distinct black triangular mark	***Aleiodes euproctis* He & Chen,1990**
–	Antenna uniformly dark brown; notauli narrow and shallow; body dark brown to reddish-brown; second metasomal tergite without black triangular mark	***Aleiodes apiculatus* Fahringer, 1932**
3	Hind coxa distinctly shorter than first metasomal tergite (0.5×); pterostigma entirely dark brown; second metasomal tergite short with yellowish-brown pentagonal mark	***Aleiodes angustupterus* van Achterberg & Shaw, 2016**
–	Hind coxa nearly as long as first metasomal tergite; pterostigma dark brown with basal third pale; second metasomal tergite longer, without yellowish-brown marking	***Aleiodes jakowlewi* Kokujev, 1898**

## Supplementary Material

XML Treatment for Aleiodes angustipterus

XML Treatment for Aleiodes euproctis

XML Treatment for Aleiodes malichi
